# Clinical Application of Individualized 3D-Printed Templates in the Treatment of Condylar Osteochondroma

**DOI:** 10.3390/healthcare10112163

**Published:** 2022-10-29

**Authors:** Wen Ma, Shiwei Niu, Lidong Wang, Canbang Peng, Shuai Fu, Changbin Zhang, Qingying Cui, Sihang Wang, Ming Li, Yanhua Xu

**Affiliations:** 1 Department of Oral and Maxillofacial Surgery, Kunming Medical University Affiliated Stomatological Hospital, Yunnan Key Laboratory of Stomatology, Kunming 650106, China; 2 Yunnan Key Laboratory of Stem Cell and Regenerative Medicine, Science and Technology Achievement Incubation Center, Kunming Medical University, Kunming 650500, China; 3 Department of Orthodontics, Kunming Medical University Affiliated Stomatological Hospital, Yunnan Key Laboratory of Stomatology, Kunming 650106, China

**Keywords:** condylar osteochondroma, virtual design, 3D-printing, templates

## Abstract

Background: Osteochondroma (OC) is one of the most common benign tumors of the long bones, but it rarely occurs in the maxillofacial skeleton. However, mandibular condylar OC often leads to severe facial deformity in affected patients, including facial asymmetry, deviation of the chin, and malocclusion. This study aimed to explore the clinical application of individualized 3D-printed templates to accurately and effectively treat condylar OC. Methods: A total of 8 patients with mandibular condylar OC were treated from July 2015 to August 2021. The enrolled patients (5 women and 3 men) had a median age of 27 years (range: 21–32 years). All patients exhibited symptoms of facial asymmetry and occlusal disorders preoperatively. The digital software used to virtually design the process consisted of three-dimensional reconstruction, 3D-cephalometry analysis, virtual surgery, individualized templates, and postoperative facial soft-tissue prediction. A set of 3D-printed templates (DOS and DOT) were used in all cases to stabilize the occlusion and guide the osteotomy. Then, pre- and post-operative complications, mouth opening, clinical signs, and the accuracy of the CT imaging analysis were all evaluated. All the measurement data were presented as means ± SD; Bonferroni and Tamhane T2 multiple comparison tests were used to examine the differences between the groups. Results: All patients healed uneventfully. None of the patients exhibited facial nerve injury at follow-up. In comparing the condylar segments with T0p and T1, the average deviation of the condylar segments was 0.5796 mm, indicating that the post-operative reconstructed condyles showed a high degree of similarity to the reconstruction results of the virtual surgical plan. Conclusions: Individualized 3D-printed templates simplified surgical procedures and improved surgical accuracy, proving to be an effective method for the treatment of patients with slight asymmetric deformities secondary to condylar OC.

## 1. Introduction

In recent years, computer-aided surgery (CAS) in the field of oral and maxillofacial treatment and the virtual planning of craniofacial surgical procedures are becoming increasingly popular [1,2,3]. Individualized 3D-printed templates and models, as an important computer-assisted surgery technology, is often applied for preoperative simulation, osteotomy, and occlusal reconstruction [2,4].

Osteochondroma (OC), also known as osteocartilaginous exostosis, is one of the most common benign tumors of the long bones, but is rarely found in the facial skeleton [5]. However, patients with mandibular condylar OC often go on to experience severe facial deformity, such as facial asymmetry, vertical elongation of the face, lateral open bite on the affected side, prognathic deviation of the chin, and temporomandibular joint (TMJ) dysfunction [6,7,8,9,10,11]. The classification of condylar OC is complex, and currently, there is no unanimous classification criteria. Min-jie Chen’s team [12] reported that there are two types of condylar OC: type 1 (protruding expansion) and type 2 (globular expansion). Type 2 OC exhibits giant mass, and the deformity is mainly presented at the mandible, along with chin deviation and lateral crossbite. Type 1 OC leads to slight deformity, and local resection of the mass is adequate. Wolford’s condylar hyperplasia classification reports CH Type 1,2,3, and 4, with various CH pathologies, as well as different clinical and imaging characteristics. The CH Type 2 (2A and 2B) is the most common type occurring with osteochondroma, resulting in a unilateral vertical over-growth deformity of the jaws; however, the horizontal growth vector can occasionally occur [13,14]. Strictly speaking, all cases in our study were exophytic tumor extensions from the condyle in the antero-medial and posterior-medial growth direction, mainly leading to a horizontal growth vector, with slight deformity in the vertical direction. 

With severe deformity, additional orthognathic correction and orthodontic treatment are needed [15,16,17]. Currently, the more consistent view for treating OCs is the excision of the tumor and the maximal preservation of some or all of the condylar head [18]. However, the condylar tumor resection process is a difficult surgical procedure, owing to the complexity of the approach and the proximity to vital anatomic structures [19]. 

Considering the problems mentioned above, for patients with slight asymmetric deformities secondary to condylar OC, the individualized 3D-printed templates contain a 3D-printed occlusion splint (DOS) and 3D-printed osteotomy template (DOT), manufactured to simplify surgical procedures and improve accuracy. Moreover, a preliminary evaluation is made to investigate the accuracy and practicability of the procedure. This novel method provides an option for the treatment of slight asymmetric deformities secondary to condylar OC.

## 2. Materials and Methods

### 2.1. Patients

From July 2015 to August 2021, 8 patients with mandibular condylar OC were treated at the Department of Oral and Maxillofacial Surgery in the Kunming Medical University School and Hospital of Stomatology. The enrolled patients (5 women and 3 men) had a median age of 27 years (range 21–32 years). All patients had symptoms of facial asymmetry and occlusal disorders preoperatively. The study was approved by the Medical Ethics Committee of Kunming Medical University School and Hospital of Stomatology (KYKQ2021MEC041).

### 2.2. Three-Dimensional Reconstruction of Bone, Facial Soft Tissue, and Vascular Bundle

**Figure 1:**
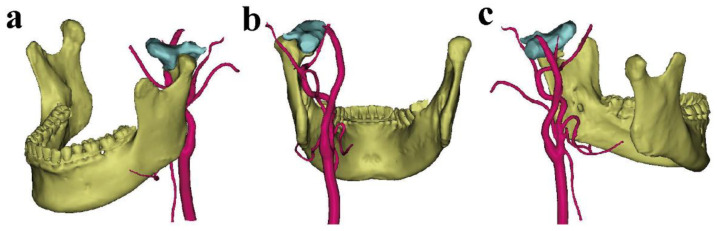
The different views of 3D reconstruction of the mandible, condylar OC, and vascular bundle: (**a**) mean lateral view; (**b**) mean posterior view; (**c**) mean medial view. (The mandible is shown in yellow, the condylar OC in light blue, and the vascular bundle in red).

The DICOM data from spiral computer tomography (CT) imaging (slice thickness 0.625 mm) were collected and transferred to Mimics 21.0 software (Materialise, Belgium) for 3D reconstruction. The surface data for teeth and surrounding soft tissue was obtained by a 3D scanner (EinScan Pro 2X, Shinning3d, Hangzhou, China). The Thresholding function, with 231 to 3050 HU and−303 to 3376 HU, was used to segment the different hard tissues and facial soft tissue. Then, the Region Growing function was performed through the coronal, sagittal, and axial windows to select the region required by the surgeon. The Edit Mask and Multiple Slice Edit tools were also used to distinguish the vascular bundle of the affected side. The Calculate Part option from the Mask function was also accessed to generate corresponding 3D models with different colors. These models showed the mandible, condylar tumor, and vascular bundle (Figure 1).

### 2.3. 3D Cephalometric Analyses

**Figure 2:**
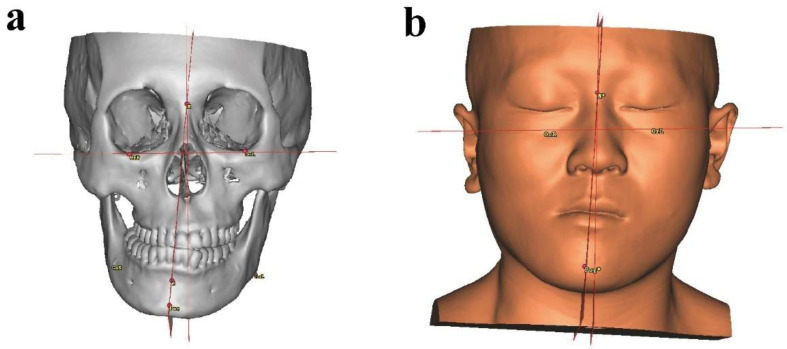
The 3D cephalometric measurements shown on virtual models: (**a**) craniomaxillofacial bone measurements; (**b**) facial soft-tissue measurements.

The maxillary and mandibular composite models were matched using bone tissue and 3D scanned tooth models, aligned into a common coordinate system, and then the facial soft-tissue model was also imported into ProPlan CMF 3.0 software (Materialise, Belgium). The measurements presented in our study were similar to those obtained using the method described by Ma, Zhigui [20]. The measurement system was described as follows: (1) The Frankfort horizontal (FH) plane was defined through the right and left orbitale (Or) and the midpoint of the bilateral porion (Po); (2) The midsagittal reference plane was defined as the plane perpendicular to the horizontal plane passing through the nasion and sella, and then adjusted through the mirror function; (3) The B, Go, Po, Pog, and Pog* points were marked on each side (Figure 2). The definitions of the measurements used in this study are listed in Table 1.

### 2.4. Virtual Surgery and Individualized 3D-Printed Template Design

**Figure 3:**
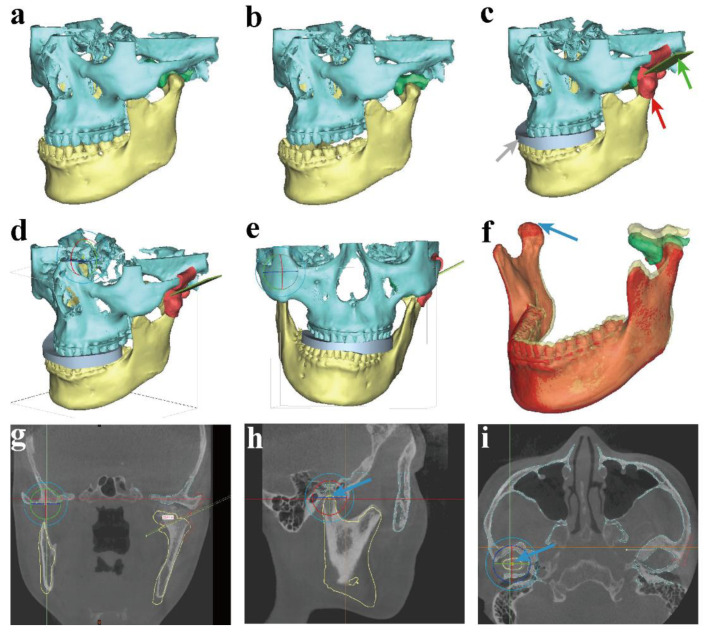
The process of virtual planning and 3D-printed template design. (**a**) The original maxilla, mandible, and condylar OC; (**b**,**c**) designed DOS, DOT, and adjust direction of the osteotomy plane in the position of the rotated mandible; (**d**–**f**) the co-point of the non-OC-affected side was set as the rotation center to roll the mandible in a 3D view; (**g**–**i**) rotation center in 2D view and measurements of osteotomy depth (gray arrow—DOS; red arrow—DOT; green arrow—osteotomy plane; blue arrow—rotation center).

In order to clearly expose the osteotomy boundary of the condyle and the OC, the mandible was held stable in the position at which the Co point of the non-OC-affected side was set as the rotation center downward to the affected side of condyle and OC with a 2.7° roll. In this occlusion, the DOS was designed to fit the upper and lower teeth. In consideration of protecting the base of the skull and the significant surrounding anatomical structures, the osteotomy plane was performed and adjusted to the most appropriate position. Then, the DOT was design using the Mark region, Extrude tool, and the Boolean operation function, and embraced with a partial condyle and root of zygomatic arch. No holes were designed in the DOT, owing to the stable mandible with the DOS used, as well as the stationary zygomatic bone. Therefore, the DOT was seated steady and easily, guiding the osteotomy precisely along the designed direction. Then, the depth of osteotomy from the DOT surface to end of the OC was measured to guide the surgery (Figure 3). All the templates were 3D printed using the SLA technique (Form 3BL, Formlabs Inc., Somerville, MA, USA) with BioMed White Resin, and the models were manufactured using FDM 3D printing (Guider 2, Flashforge Co., Ltd., Zhejiang, China) with PLA material. Then, all the 3D printing templates had to be attached smoothly and firmly to the models before the operation. The final models were used in surgery after disinfection using low-temperature plasma.

### 2.5. Facial Morphology Simulation

The mandible and chin were deviated to the non-OC-affected side before surgery, and the facial morphology was also deviated owing to the existence of OC. When the OC was removed during virtual surgery, the affected side condyle went back to the articular fossa, according to final occlusion. The maxilla was not changed in this position; however, the mandible and chin were moved to near the midsagittal plan; thus, the facial morphology was also changed. Therefore, the soft tissue simulation was performed to predict facial morphology after OC removal in virtual surgery. The video is available in the Supplement Files (Video S1).

### 2.6. Surgical Procedures

**Figure 4:**
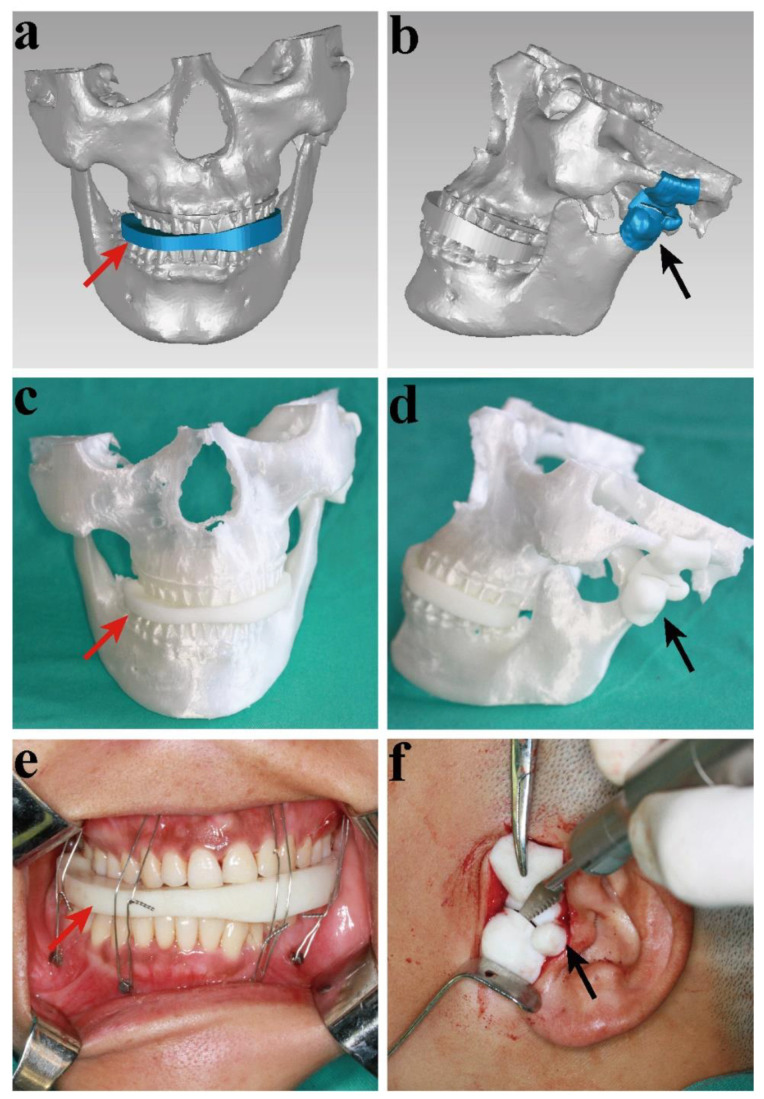
The virtual and 3D-printed models of the surgery: (**a**) The virtual 3D-printed occlusion splint (DOS); (**b**) the virtual 3D-printed osteotomy template (DOT); (**c**) the DOS and 3D-pring models; (**d**) the DOT and 3D-pring models; (**e**) the DOS in surgery; (**f**) the DOT in surgery (red arrow—DOS; black arrow—DOT).

Surgeries were performed under general anesthesia through nasal endotracheal intubation. The L shaped pretragal incision was made, incising the tissue in layers to expose the condylar surface. According to the preoperative plan, the DOS was first used to fit the occlusion with a wire fixation; in this step, an adequate dose of muscle relaxants was required. When the osteotomy boundary was clearly exposed, the DOT was located in the steady joint space. The osteotomy line was obtained using a reciprocating saw using the 3D-printed template, through which the direction and measured osteotomy depth were determined preoperatively. Then osteotome and round drill were used to remove the condylar OC and trim the edge of osteotomy (Figure 4). When the tumor with the affected condyle was completely removed, the DOS could be removed. No remarkable bleeding was observed; however, a suction drain was inserted, and the wound was closed in layers. Elastic traction, with bone screws in the maxilla and mandible alveolar structures, was needed postoperatively to achieve the normal occlusal relationship.

### 2.7. Postoperative Validation and Follow-Up

All patients underwent a craniomaxillofacial CT scan at 1 week after surgery (T1) and 1 year (T2) postoperatively. The (T0) shows the CT scan before surgery, and the (T0p) CT data shows virtual preoperative plan. All patients were evaluated by clinical check, accuracy evaluation, 3D cephalometry, and TMJ space measurements in during the follow-up period.

#### 2.7.1. Clinical Evaluations

All patients were evaluated ragarding facial morphology, swelling, symptoms of TMJ (clicking and noise, pain, and movement), and occlusion during the follow-up period.

#### 2.7.2. Accuracy Evaluation of DOS and DOT

In order to evaluate the accuracy of the DOS and DOT in the surgery process, the actual postoperative operation CT data (T1) and the virtual plan (T0p) were imported in Mimics software, and the deviations were measured using Geomagic Studio 2014 software (Raindrop, Wilmington, NC, USA). The postoperative condyle segment was registered to the planned models, then chromatography was used to evaluate the differences between them. At last, quantitative error analysis was carried out. 

#### 2.7.3. 3D Cephalometry

The (T0, T1, T2) CT data were imported in Proplan CMF (Materialise, Belgium), landmarks were drawn in 2D and 3D imaging, and linear and angular measurements were obtained in all 3 planes on 3D models, according to cephalometry measurements, as presented in Figure 2.

#### 2.7.4. TMJ Space Measurements

To evaluate the change of condylar position in different periods (T0p, T1, T2), the linear measurements of the OC joint space in the actual postoperative and virtual planned models were assessed and compared with each other. All linear vectors were defined from the most prominent condylar points (anterior, superior, posterior, medial, lateral) to the glenoid fossa, in accordance with a previous study [20,21]. The anterior joint space (AJS), superior joint space (SJS), and posterior joint space (PJS) were measured in the sagittal image, and the medial joint space (MJS) and lateral joint space (LJS) were measured in the coronal image.

### 2.8. Statistical Analysis

All measurement data are presented as means ± S.D., and Bonferroni and Tamhane T2 multiple comparison tests were used to examine the differences between the groups. The Shapiro–Wilk normality test and Levene’s variance homogeneity test were also applied to the data. All statistical analyses were conducted using SPSS software version 26.0 (SPSS Inc., Chicago, IL, USA). Statistical significance was set at *p* < 0.05.

## 3. Results

**Figure 5:**
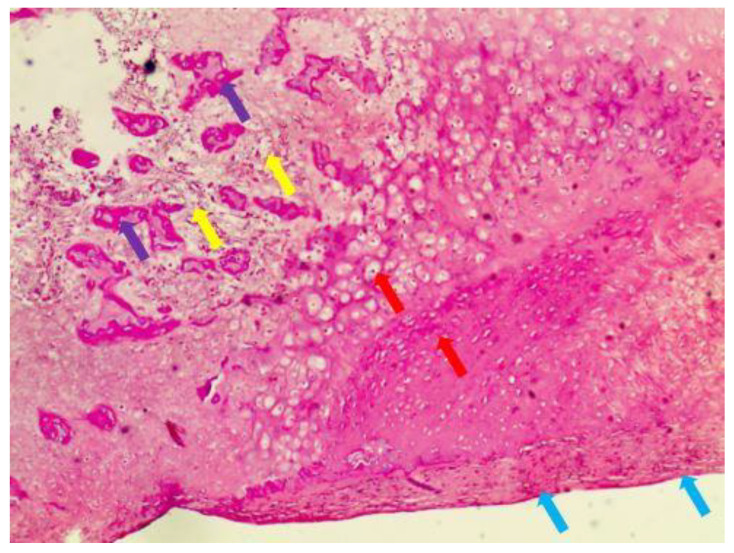
Hematoxylin and eosin (H&E) staining (yellow arrows—yellow marrow in the lesion; red arrows—hyaline cartilage cap with columns of cartilage cells; purple arrows—trabeculae within the osteochondroma; blue arrows—fibrous capsule or perichondrium).

A total of 5 females and 3 males with condylar OC were included in the study. The average age of the patients was 27 years old. The condylar OC resection and conservative condylectomy were successfully carried out using DOS and DOT in all patients. The diagnosis of condylar OC was verified by a postoperative pathological examination (Figure 5). Under microscopic examination, the cartilage cap appeared to merge with the underlying bone, and it was covered with a thin layer of fibrous capsule that functions as a perichondrium. The cartilage cap resembled a growth plate, with columns or clusters of chondrocytes evenly distributed and maturing in an enchondral process. The process of enchondral ossification led to medullary bone, typically with yellow rather than hematopoietic marrow, and large calcified areas or calcific debris. All examinations were conducted as follows.

### 3.1. Evaluation of Clinical Features

**Figure 6:**
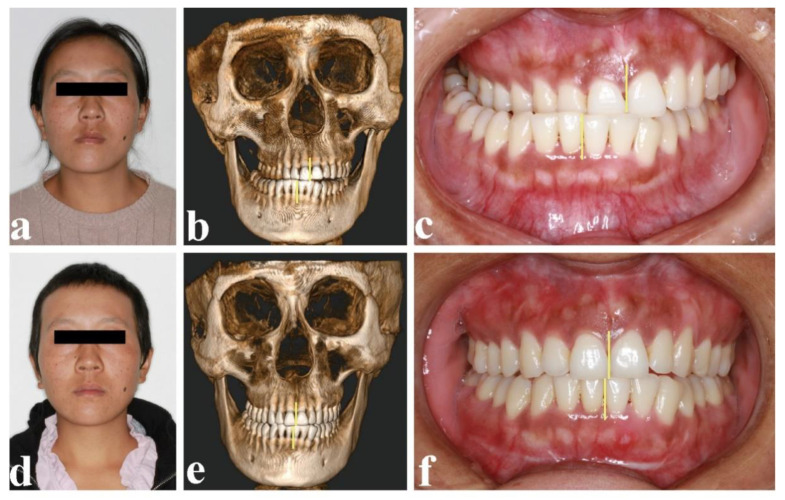
The change of facial profile, 3D CT image, and occlusion preoperatively and 1 year after surgery: (**a**,**d**) frontal profile, preoperatively and postoperatively; (**b**,**e**) frontal 3D CT image, preoperatively and postoperatively; (**c**,**f**) intraoral view, preoperatively and postoperatively (yellow line—middle line of upper and lower jaw).

**Figure 7:**
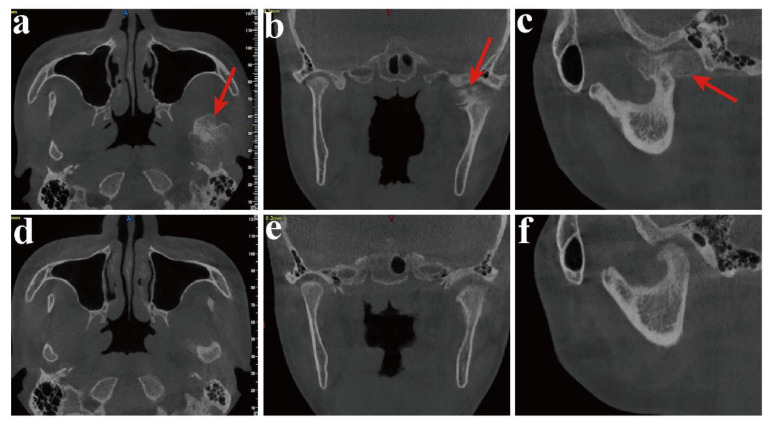
The change in the 2D CT image preoperatively and 1 year after surgery: (**a**,**d**) axial CT scan,, preoperatively and postoperatively; (**b**,**e**) coronal CT scan, preoperatively and postoperatively; (**c**,**f**) sagittal view, preoperatively and postoperatively (red arrow—condylar OC).

None of the patients experienced complications, including facial nerve injury or salivary fistula. The symptoms of facial deviation and malocclusion were improved (Figure 6). There was no significant difference in mouth opening before (28.7 mm) and after surgery (32.4 mm). The patients showed no signs of tumor recurrence or TMJ ankylosis during an average follow-up of 20.4 months (Table 2). Radiological examination was part of the follow-up routine. The location and morphology of the condyle were evaluated (Figure 7). The CBCT (cone beam CT) at 1 week after surgery revealed that the 3D printing DOS and DOT accurately transferred the simulated operation to the actual operation.

### 3.2. Evaluation of the Accuracy of Condylar Osteotomy with DOS and DOT

**Figure 8:**
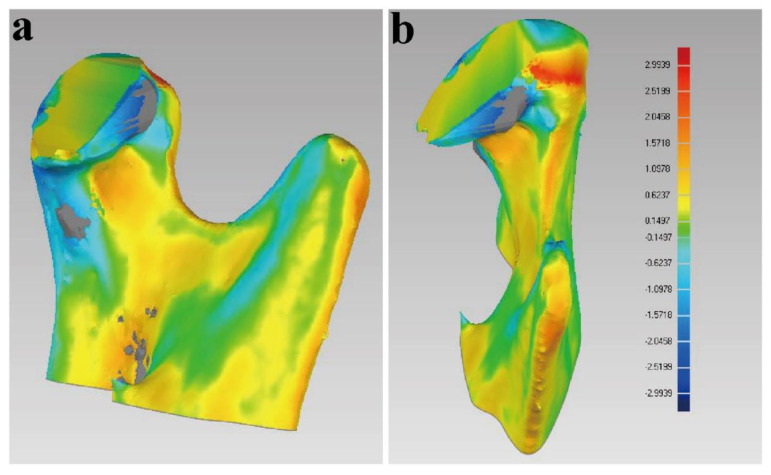
Evaluation of the accuracy of DOS and DOT: (**a**,**b**) superimposition of the virtual condylar segment and the postoperative CT scan at 1 week after surgery, for accuracy analysis.

For the comparison of the condylar segments with (T0p) and (T1), the results were displayed on a color-coded discrepancy map. Fifteen color segments were selected, and the maximum critical value to minimum value was from 2.9939 mm to −2.9939 mm. The 3D deviations between (T0p) and (T1) are shown in Table 3. The average deviation of condylar segments was 0.5796 mm, and the standard deviation was 0.6774 mm. The most obvious area of change was in the osteotomy plane, owing to the fact that bone needed to be trimmed during the operation. The maximum deviation of buccal, lingual, mesial, and distal sides were 1.11 mm, 1.15 mm, 1.34 mm, and 1.17 mm, respectively, indicating that the postoperative reconstructed condyles (T1) had a high degree of similarity to the reconstruction shown in the virtual surgical planning (T0p) (Figure 8).

### 3.3. Changes on 3D Cephalometric Analyses 

The 3D cephalometric measurements at T0, T1, and T2 are shown in Table 4. In all liner measurements, the distance of the Go to FH on the OC-affected side, and the B, Pog, and Pog* points to the SP plane on the non-OC affected side, were statistically significantly reduced (*p* < 0.05). The deviation angles of the B and Pog* points to the SP plane were also decreased postoperatively (*p* < 0.05). These results showed that facial deformity was corrected in both the bone and the soft tissue.

### 3.4. Changes in Joint Space 

The joint space measurements of the 2 sides in T0p, T1, T2 are shown in Table 5. On the unaffected side, all joint spaces showed slight changes in different time periods. On the OC-affected side, the AJS, SJS, and PJS were decreased (T1 vs T0P), but then increased from T1 to T2. The MJS and LJS were increased from T0p to T1 and T2. This result indicated that the neo condylar segment was adaptive and seated in the proper position. 

## 4. Discussion

The traditional method to treat condylar OC is total condylectomy or local resection of the lesion, orthognathic surgery, and condylar replacement, usually with a costochondral graft or total joint prosthesis [16,17,22,23]. Currently, a conservative treatment modality, with excision of the tumor and maximal preservation of some or all of the condylar head is generally accepted [18]. The surgical methods are different depending on type of OC. In some patients with slight mandibular asymmetry and malocclusion, local resection of the mass, with the condylar head preserved, not only solves the malocclusion, but also corrects the facial asymmetry, which is of a great benefit for facial morphology [12].

The intraoral or extraoral approach have been reported in the treatment of OC [15,24,25,26,27]. Yu, H B [23] reported the application of an endoscope to treat condylar OC through an intraoral approach, which proved to an effective method of treatment. Other studies claimed that the use of navigation technology in an intraoral approach could also be used in the treatment of condylar OC; the advantages of this method were safety, precision, and time savings [5]. Although the researchers reported several advantages of navigation and endoscopic technology in the treatment of the condylar OC, there were still some deficiencies, such as the limited surgical field exposure, high expense, difficulty of use, the invasiveness of the operation to place the receiver, registration error, etc. [28]. In the extraoral approach, the surgical region was exposed more clearly than in the intraoral method; although this incision has the risk of injury to the facial nerve, the incidence of complete injury was very low [25]. Two factors leading to the resection of the local exophytic OC with the condylar head remained difficult. One difficult was the viewing of the stalk of the OC region; when the tumor was localized in the medial side of condyle, the surgeon could not see the stalk of the OC owing to the narrow space from the condyle to the articular fossa created by the extraoral incision. Another problem was the mandible position, as the stabilization of the mandible was necessary when osteotomy was performed, or the incorrect osteotomy line, as well as soft tissue injury, would occur. In considering the above, the problem of stabilizing the mandible while exposing the stalk of the OC for osteotomy were the most urgent problems to solve.

With the development of computer-aided surgical (CAS) simulation, it became possible to successfully transfer preoperative design schemes to intraoperative plans [29]. This method allowed oral and maxillofacial surgeons to overcome most of these limitations. Computer-aided surgery included 3D reconstruction, virtual surgery, mirror techniques, 3D-printed template design, and so on. It was essential to use these techniques to formulate individual surgical plans with patients suffering from condylar OC. Ord, R A [30] reported that preoperative planning was extremely significant in the treatment of condylar OC. Preoperative designing of digital templates could make surgery more accurate and safe. A variety of programs could be used for the preoperative design in order to save time during the operation [31]. Lu, C [32] claimed that the digital occlusal splint exhibited some advantages, including a shorter treatment time in temporomandibular joint surgery.

In our study, all 8 patients who accessed DOS and DOT during their treatment for condylar OC lead to a horizontal growth vector, achieving satisfactory results. In the surgical process, the DOS was located between the upper and lower teeth, fixed with wires, while the affected side condyle, and OC rotated to clearly expose the stalk of the osteotomy region. The mandible was stabilized by DOS; then the DOT was located in the position designed preoperatively to guide osteotomy accuracy in regards to depth and direction. In consideration of protecting the base of the skull and the significant surrounding anatomical structures, an osteotomy plane was created and adjusted to the most appropriate position. Furthermore, the stable mandible transmitted the force to the stalk of OC, preventing slippage and injury to the important anatomical structures when the saw and chisel were in use.

During the treatment, there are three major possible complications, including facial paralysis, massive bleeding during operation, and cranial base injury [21,25]. However, the virtual plan and 3D-printed templates can decrease the occurrence of such complications [21].

First of all, the surgeon could preoperatively determine the relationship between the blood vessel and the condylar OC in the 3D view to avoid the injury to important anatomical structures. Furthermore, the condylar OC was often located in the medial of the condyle, and intraoperative mandible movement resulted in difficulty of the osteotomy. Thus, 3D-printed templates could solve this problem. The osteotomy direction and depth were designed before operation to protect the cranial base and avoid injury to blood vessels located in the medial of the condylar OC. The safe distance could help control the depth of osteotomy in order to protect vascular structures. As for the neural structures, sometimes, during the process of cutting, a facial nerve is injured by accidental saw slippage. The DOT can fix the saw, avoiding injury to the facial nerve. In addition, the complication of facial paralysis should be considered. Although some studies reported that extraoral incision could easily damage the facial nerve, there were no facial nerve injuries in our study. There are many causes for facial paralysis; in addition to stretch injury, the reason for paralysis may due to the lack of constraint of the osteotomy tool, causing slippage and direct injury of the facial nerve. Such complications could be avoided by using of the DOT, owning to the high edge of the plate and control of the osteotomy direction. Preoperative digital design, including depth-limiting and precise measurement, ensured that the osteotomy did not extend too deeply or deviate, completely avoiding serious life-threatening complications, such as accidental cranial base injury or injury to the internal maxillary artery or pterygoid plexus.

The facial morphology simulation, which is widely used in orthognathic surgery, provided surgeons and patients with a better soft tissue prediction before treatment [33,34,35]. However, this simulation method is less reported in condylar OC patients. In our study, we tried to use soft tissue prediction to simulate the change in facial morphology after virtual surgery, as shown in the Supplement Files (Video S1). The patient could visualize the video to see how the mandible would be moved after surgery and how the face profile would change, enabling better communication between patients and surgeons. The change in facial morphology simulated in the software not only helped surgeon to evaluate the asymmetry of the facial morphology after surgery, but also enabled the convenient communication of the procedure to the patients. 

From the 3D cephalometric analyses, the liner vectors and deviated angles were measured at different periods. The distance of the Go point to the FH plane was significantly decreased in the OC-affected side, with nearly no change in normal side, indicating that the enlarged condyle segment was corrected. Furthermore, the distances of the B, Pog, and Pog* points to the SP plane were changed significantly, with average data showing movement of about 8mm. Furthermore, the B point deviated angles changed from 5.73° to 1.93° and 1.14°, on average, and the Pog* deviated angle also changed from 5.78° to 0.94° postoperatively. All the liner vector results showed that the facial deviation improved, which was consistent with the deviated angles observed. The evaluation of the 3D cephalometric analyses helped surgeons estimate the change in skeleton and facial tissues in different periods.

In the present study, the superimposition of the planned models and postoperative condyle segment showed that the mean 3D deviation of the condylar osteotomy guide between T0p and T1 was 0.5794 mm, which was similar to that in Lei Qi’s study [21]. The maximum deviation was 1.34 mm, with average in lingual sides owing to the need to grind the surface of the neo condyle smooth when the osteotomy performed. The results proved that the DOS and DOT transformed the virtual plan into a real operation, improving the precision of the osteotomy.

In view of condylar position postoperatively, the joint spaces of the different sides were measured. From T0p to T1, the space results for both the OC-affected and unaffected sides changed only slightly, indicating that the designed virtual plan was reasonable, and that the DOS and DOT provided accurate surgical guides. The measurements were changed from T1 to T2, showing the adaptive reconstruction process of the neo condyle. In the two sides, the AJS, SJS, PJS, and MJS vectors were increased in our study; however, these results were the opposite of those obtained in the study of Zhigui Ma, which showed decreases in AS and SS in OC-affected and unaffected sides [20]. This may be due to the different postoperative orthodontic methods used. The change tendency of the joint space was similar to that observed in a previous study combining condylectomy and orthognathic surgery to treat condyle OC [21]. The results showed that the joint space continued to change slightly to adapt to the neo condyle position, and the clinical examination of the condyle function showed normal results at the follow-up. The postoperative clinical features and radiological examination results indicated that all the patients healed uneventfully when the 3D-printed templates were used in the treatment of condylar OC.

## 5. Conclusions

In short, in the future, the tendency will be to integrate medicine and engineering. Our research aimed to evaluate the application of individualized 3D-printed templates in the treatment of slight asymmetric deformities secondary to condylar OC. The tumors were precisely removed using DOS and DOT, and the neo condylar position was adaptively reconstructed, with no other complications. Therefore, the virtual design and 3D-printed template methods used to treat condylar OC with slight deformity exhibit the advantages of predictability, more precise osteotomy, and trauma reduction. This innovative method can provide a valuable treatment option for this complicated procedure in the future.

## Figures and Tables

**Table 1:** Definitions of the 3D cephalometric landmarks and measurements.

**Landmarkers**	**Definition**
Orbitale (Or)	The most inferior point on margin of the orbit
Porion (Po)	The most superior point of the outline of the external auditory meatus
Codyle (Co)	The most superior point of the condyle
Nasion (N)	The midpoint of the frontonasal suture
Nasion of soft tissue (N*)	The soft tissue nasion
Sella (S)	The center of the hypophyseal fossa (sella turcica)
Gonion (Go)	The most caudal and most posterior point of the mandibular angle
B point	The most concave point on the mandibular symphysis
Pogonion (Pog)	The most anterior midpoint of chin
Pogonion of soft tissue (Pog*)	The most anterior midpoint of soft chin
FH plane	The plane defined by point OrL, OrR, and point Mid Po
Midsagittal plane (SP)	The plane through point S, point N, and the normal plane FH, with mirror function
**Measurement**	**Definition**
Go-FH, mm	The shortest distance between the gonion and the Frankfort plane
Go-SP, mm	The shortest distance between the gonion and the SP
B-SP, mm	The shortest distance between the B and the SP
Pog-SP, mm	The shortest distance between the Pog and the SP
Pog*-SP, mm	The shortest distance between the Pog* and the SP
B deviated angle, °	The angle between the SP and the B-nasion vector
Pog* deviated angle, °	The angle between the SP and the Pog*-soft tissue nasion vector

FH = Frankfort horizontal; SP = midsagittal plane.

**Table 2:** Patient characteristics.

Case No.					Mouth Opening (mm)		
	Age (Years)	Gender	Affected Side	Facial Nerve Injury	Preop.	Postop.	Follow-Up (Months)
1 2 3 4 5 6 7 8	23 21 27 25 29 31 28 32	F M F M F F F M	L R L L L R R L	No No No No No No No No	29 27.5 28.5 31 29.5 28.5 27 28.5	33 31 32 33 34.5 31 32 33	20 14 24 19 30 18 25 13
Average	27	-	-	-	28.7	32.4	20.4

M, male; F, female; L, left; R, right; Preop., preoperative; Postop., postoperative.

**Table 3:** Postoperative accuracy study of osteotomy in 3D deviation (mm) with T1 vs T0p.

Patients	Critical Range (mm)	Max DV (Buccal)	Max DV (Lingual)	Max DV (Mesial)	Max DV (Distal)	Mean DV	Standard DV
1	2.99~−2.99	1.01	0.98	1.52	1.04	0.4988	0.6401
2	2.99~−2.99	1.21	1.14	1.75	1.22	0.6254	0.7041
3	2.99~−2.99	0.91	1.21	1.46	1.31	0.6180	0.6956
4	2.99~−2.99	1.05	1.19	1.24	0.97	0.5262	0.6182
5	2.99~−2.99	0.94	1.29	1.02	1.34	0.5715	0.6529
6	2.99~−2.99	1.15	1.22	1.38	1.27	0.6526	0.7251
7	2.99~−2.99	1.33	1.04	1.15	1.08	0.5420	0.6624
8	2.99~−2.99	1.28	1.16	1.21	1.09	0.6024	0.7211
Average	-	1.11	1.15	1.34	1.17	0.5796	0.6774

Max = Maximum; DV = deviation; T0p = preoperative virtual plan; T1 = 1 week after surgery.

**Table 4:** Comparisons of 3D cephalometric measurements at T0, T1, and T2.

	T0	T1	T2	*p* Value	Post Hoc Comparisons
**OC-affected side**					
Go-FH, mm	69.81 ± 2.82	64.5 ± 2.8	62.79 ± 2.26	0.001	T0 > T1, T2
Go-SP, mm	39.23 ± 2.49	39.43 ± 2.68	40.24 ± 2.57	0.712	-
**Non-OC affected side**					
Go-FH, mm	62.38 ± 2.2	62.99 ± 1.91	63.1 ± 2.06	0.754	-
Go-SP, mm	44.86 ± 2.63	41.75 ± 2.59	41.75 ± 2.48	0.053	-
B-SP, mm	9.73 ± 0.83	4.26 ± 0.68	1.4 ± 0.31	0.001	T0 > T1 > T2
Pog-SP, mm	10.34 ± 0.64	4.52 ± 0.81	1.51 ± 0.34	0.001	T0 > T1 > T2
Pog*-SP, mm	10.14 ± 0.62	4.33 ± 0.8	1.43 ± 0.28	0.001	T0 > T1 > T2
B deviated angle, °	5.73 ± 0.68	1.93 ± 0.35	1.14 ± 0.37	0.001	T0 > T1 > T2
Pog* deviated angle, °	5.78 ± 0.57	1.45 ± 0.31	0.94 ± 0.39	0.001	T0 > T1, T2

FH = Frankfort horizontal; SP = midsagittal plane; OC = osteochondroma; T0 = pretreatment; T1 = 1 week after surgery; T2 = 1 year after surgery.

**Table 5:** Different joint space measurements at T0p, T1, and T2.

	T0p (Mean ± SD)			T1 (Mean ± SD)			T2 (Mean ± SD)		
	OC-Afs	Ufs	Dif	OC-Afs	Ufs	Dif	OC-Afs	Ufs	Dif
AJS	2.23 ± 0.28	1.84 ± 0.18	0.39 ± 0.34	1.75 ± 0.24	1.56 ± 0.41	0.19 ± 0.41	2.53 ± 0.43	1.75 ± 0.24	0.78 ± 0.58
SJS	3.99 ± 0.31	2.41 ± 0.2	1.58 ± 0.24	3.93 ± 0.38	2.95 ± 0.24	0.98 ± 0.37	4.26 ± 0.67	3.36 ± 0.27	0.9 ± 0.92
PJS	4.83 ± 0.25	1.65 ± 0.24	3.18 ± 0.15	4.46 ± 0.82	2.15 ± 0.24	2.31 ± 0.98	4.81 ± 0.72	2.56 ± 0.43	2.25 ± 0.99
MJS	7.05 ± 0.24	4.6 ± 0.32	2.45 ± 0.36	8.41 ± 1.04	4.76 ± 0.27	3.65 ± 1.28	9.28 ± 0.73	5.93 ± 0.36	3.35 ± 0.78
LJS	3.41 ± 0.29	2.69 ± 0.34	0.73 ± 0.15	4.19 ± 0.63	2.94 ± 0.27	1.25 ± 0.79	4.19 ± 0.71	2.78 ± 0.28	1.41 ± 0.97

Dif = difference; Afs = affected side; Ufs = unaffected side; T0p = preoperative virtual plan; T1 = 1 week after surgery; T2 = 1 year after surgery; AJS = anterior joint space; SJS = superior joint space; PJS = posterior joint space; MJS = medial joint space; LJS = lateral joint space; SD = standard deviation.

## Data Availability

The data presented in this study are available on request from the corresponding author.

## Supplementary Materials

The following supporting information can be downloaded at: https://www.mdpi.com/article/10.3390/healthcare10112163/s1, Video S1: Facial morphology simulation.
